# Dichloridobis(*N*,*N*′-diethyl­thio­urea-κ*S*)mercury(II)

**DOI:** 10.1107/S1600536810028825

**Published:** 2010-07-24

**Authors:** Muhammad Mufakkar, M. Nawaz Tahir, Haseeba Sadaf, Saeed Ahmad, Abdul Waheed

**Affiliations:** a Department of Chemistry, Government College University, Lahore, Pakistan; b Department of Physics, University of Sargodha, Sargodha, Pakistan; c Department of Chemistry, University of Engineering and Technology, Lahore 54890, Pakistan

## Abstract

There are two mol­ecules in the asymmetric unit of the title compound, [HgCl_2_(C_5_H_12_N_2_S)_2_]. In both mol­ecules, the *N*,*N*′-diethyl­thio­urea ligands exhibit a *cis*,*trans* geometry around their C—N amide bonds. The shapes of the mol­ecules are, to a large extent, determined by intra­molecular N—H⋯Cl hydrogen bonds formed by the N—H groups from the *cis* amide groups. In one mol­ecule, these groups are involved in three-center hydrogen bonds involving both chloride ligands, whereas in the other mol­ecule only one Cl ligand takes part in intra­molecular hydrogen bonding. The coordination around the Hg atom is distorted tetra­hedral with an S_2_Cl_2_ donor set. Inter­molecular hydrogen bonds between N—H groups from the *trans* amide units of the thio­amide ligands and the chloride ligands connect the mol­ecules into a polymeric chain extending along the *c* axis. One of the ethyl groups of the *N*,*N*′-diethyl­thio­urea ligands is disordered over two positions in one of the mol­ecules, with an occupancy of 0.654 (17) for the major component.

## Related literature

For the complexation of various thio­nes with *d* ^10^ metal ions, see: Isab *et al.* (2002 ▶); Ahmad *et al.* (2009 ▶); Hanif *et al.* (2007 ▶); Mufakkar *et al.* (2009 ▶). For a related structure, see: Stalhandske *et al.* (1997 ▶): For graph-set notation, see: Bernstein *et al.* (1995 ▶).
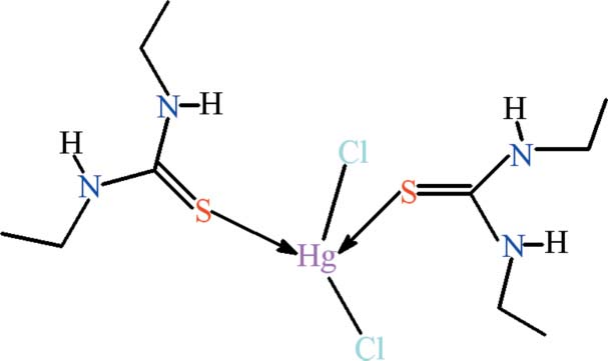


## Experimental

### 

#### Crystal data

[HgCl_2_(C_5_H_12_N_2_S)_2_]
*M* *_r_* = 535.94
Monoclinic, 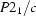

*a* = 7.9713 (2) Å
*b* = 17.2321 (5) Å
*c* = 27.5143 (7) Å
β = 94.870 (1)°
*V* = 3765.78 (17) Å^3^
*Z* = 8
Mo *K*α radiation
μ = 8.68 mm^−1^
*T* = 296 K
0.24 × 0.18 × 0.16 mm

#### Data collection

Bruker Kappa APEXII CCD diffractometer
Absorption correction: multi-scan (*SADABS*; Bruker, 2005 ▶) *T*_min_ = 0.180, *T*_max_ = 0.205
28191 measured reflections
6825 independent reflections
4529 reflections with *I* > 2σ(*I*)
*R*_int_ = 0.048

#### Refinement

*R*[*F* ^2^ > 2σ(*F* ^2^)] = 0.033
*wR*(*F* ^2^) = 0.067
*S* = 1.02
6825 reflections
353 parameters
4 restraints
H-atom parameters constrained
Δρ_max_ = 0.64 e Å^−3^
Δρ_min_ = −0.63 e Å^−3^

### 

Data collection: *APEX2* (Bruker, 2009 ▶); cell refinement: *SAINT* (Bruker, 2009 ▶); data reduction: *SAINT*; program(s) used to solve structure: *SHELXS97* (Sheldrick, 2008 ▶); program(s) used to refine structure: *SHELXL97* (Sheldrick, 2008 ▶); molecular graphics: *ORTEP-3 for Windows* (Farrugia, 1997 ▶) and *PLATON* (Spek, 2009 ▶); software used to prepare material for publication: *WinGX* (Farrugia, 1999 ▶) and *PLATON*.

## Supplementary Material

Crystal structure: contains datablocks global, I. DOI: 10.1107/S1600536810028825/gk2292sup1.cif

Structure factors: contains datablocks I. DOI: 10.1107/S1600536810028825/gk2292Isup2.hkl

Additional supplementary materials:  crystallographic information; 3D view; checkCIF report

## Figures and Tables

**Table:** 

Hg1—Cl1	2.6220 (17)
Hg1—Cl2	2.5767 (16)
Hg1—S1	2.4335 (15)
Hg1—S2	2.4323 (17)
Hg2—Cl4	2.5487 (16)
Hg2—S3	2.4415 (18)
Hg2—S4	2.4534 (14)
Hg2—Cl3	2.6046 (16)

**Table:** 

Cl1—Hg1—Cl2	91.78 (5)
Cl1—Hg1—S1	107.27 (5)
Cl1—Hg1—S2	106.50 (6)
Cl2—Hg1—S1	109.97 (5)
Cl2—Hg1—S2	105.65 (6)
S1—Hg1—S2	129.25 (5)
Cl4—Hg2—S3	110.47 (6)
Cl4—Hg2—S4	110.81 (5)
S3—Hg2—S4	123.74 (6)
Cl3—Hg2—S4	100.69 (5)
Cl3—Hg2—Cl4	98.04 (7)
Cl3—Hg2—S3	109.51 (7)

**Table 2:** Hydrogen-bond geometry (Å, °)

*D*—H⋯*A*	*D*—H	H⋯*A*	*D*⋯*A*	*D*—H⋯*A*
N1—H1⋯Cl3	0.86	2.43	3.205 (5)	150
N2—H2⋯Cl1	0.86	2.56	3.406 (5)	169
N3—H3⋯Cl3^i^	0.86	2.47	3.239 (5)	149
N4—H4⋯Cl1	0.86	2.63	3.442 (6)	159
N4—H4⋯Cl2	0.86	2.96	3.440 (6)	117
N6—H6⋯Cl4	0.86	2.44	3.299 (5)	174
N7—H7⋯Cl4	0.86	2.49	3.340 (5)	173
N8—H8⋯Cl2	0.86	2.58	3.390 (5)	158
C14—H14*B*⋯Cl2^ii^	0.97	2.82	3.699 (6)	151
C17—H17*A*⋯Cl2	0.97	2.77	3.448 (6)	127

Symmetry codes: (i) 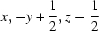; (ii) 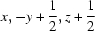.

**Fig. 1.**
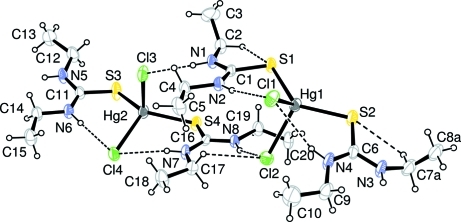
View of the title compound with the atom numbering scheme. The displacement ellipsoids are drawn at the 30% probability level. H-atoms are shown by small circles of arbitrary radii. The dotted lines show intramolecular H-bondings. Only one position of the disordered ethyl group, the atoms C7a and C8a, is shown.

**Fig. 2.**
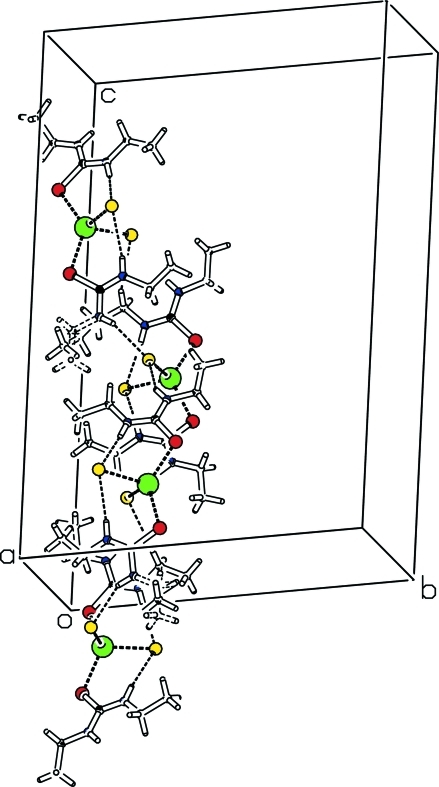
The partial packing (PLATON; Spek, 2009) which shows one dimensional polymeric chain via hydrogen bonds extending along c axis.

## supplementary crystallographic information

### Comment

We have been investigating the complexation of various thiones with d^10^ metal ions in order to assess their modes of binding and to explore the related structural and spectral properties (Isab *et al.*, 2002; Ahmad *et al.*, 2009; Hanif *et al.*, 2007; Mufakkar *et al.*, 2009). Mercury(II) is a typical soft Lewis acid and shows a specific affinity to sulfur donors such as thiones. Crystal structures of several mercury(II) complexes of thiones reveal that mercury(II) coordinates with thiones through sulfur atom in a tetrahedral or pseudoterahedral environment (Ahmad *et al.* 2009). In continuation of our efforts to study the coordination behavior of thiones towards d^10^ metal ions, we report herein the structure and synthesis of the title compound (I, Fig. 1)..

The crystal structure of (II) *i.e.*, dichloro-bis(*N*,*N*-dimethylthioformamide)-mercury(ii) (Stalhandske *et al.*, 1997) has been published which have similar coordination around the mercury atom.

The title compound consists of two molecules in the crystallographic asymmetric unit which differ from each other geometrically. In both molecules, the coordination around mercury atom is distorted tetrahedral with two S and two Cl atoms. In one molecule, the thiocyanide groups of two diethylthiourea are oriented at a dihedral angle of 23.94 (14)°, whereas in the other its value is 31.74 (16)°. In Hg1 containing molecule, the Hg—Cl and Hg—S bond distances have values of [2.5767 (16), 2.6220 (17) Å] and [2.4323 (17), 2.4335 (15) Å], respectively. In Hg2 containing molecule, the Hg—Cl and Hg—S bond distances have values of [2.5487 (16), 2.6046 (17) Å] and [2.4415 (18), 2.4534 (14) Å], respectively. In first molecule, the bond angles around Hg1 have a range of 91.78 (5)–129.25 (5)°. In the second molecule, the bond angles around Hg2 have a range of 98.04 (7)–123.74 (6)°. This shows that there is wide difference between two molecules. The important bond distances and bond angles are given in Table 1. In both molecules intramolecular H-bondings of N—H···Cl type complete two S(6) ring (Bernstein *et al.*, 1995) motifs (Table 2, Fig. 1). The molecules are stabilized in the form of infinite one dimensional polymeric chains extending along the crystallographic *c* axis due to intermolecular H-bondings of C—H···Cl and N—H···Cl types (Fig. 2).

### Experimental

To mercury(II) chloride (0.27 g, 1.0 mmol) in 10 ml of methanol was added two equivalents of *N*,*N'*-diethylthiourea in 15 ml of methanol. On mixing a clear solution was obtained that was stirred for 30 minutes. The colorless solution was filtered and the filtrate was kept at room temperature for crystallization. A white crystalline product was obtained that was washed with methanol and dried.

### Refinement

The ethyl group with the atoms C7 and C8 is disordered over two positions denoted as a and b. Four restraints were imposed on the bond lengths of this group. The displacement parameters of the C7a and C7b as well as C8a and C8b were constrained to be equal. The occupancy factor of the major position refined at 0.654 (17).

The H-atoms were positioned geometrically (N–H = 0.86, C–H = 0.96–0.97 Å) and refined as riding with *U*_iso_(H) = *xU*_eq_(C, N), where *x* = 1.5 for methyl and *x* = 1.2 for all other H-atoms.

### Crystal data

**Table:**

[HgCl_2_(C_5_H_12_N_2_S)_2_]	*F*(000) = 2064
*M**_r_* = 535.94	*D*_x_ = 1.891 Mg m^−^^3^
Monoclinic, *P*2_1_/*c*	Mo *K*α radiation, λ = 0.71073 Å
Hall symbol: -P 2ybc	Cell parameters from 4529 reflections
*a* = 7.9713 (2) Å	θ = 2.4–25.3°
*b* = 17.2321 (5) Å	µ = 8.68 mm^−^^1^
*c* = 27.5143 (7) Å	*T* = 296 K
β = 94.870 (1)°	Prism, white
*V* = 3765.78 (17) Å^3^	0.24 × 0.18 × 0.16 mm
*Z* = 8	

### Data collection

**Table:**

Bruker Kappa APEXII CCD diffractometer	6825 independent reflections
Radiation source: fine-focus sealed tube	4529 reflections with *I* > 2σ(*I*)
graphite	*R*_int_ = 0.048
Detector resolution: 8.2 pixels mm^-1^	θ_max_ = 25.3°, θ_min_ = 2.4°
ω scans	*h* = −8→9
Absorption correction: multi-scan (*SADABS*; Bruker, 2005)	*k* = −20→20
*T*_min_ = 0.180, *T*_max_ = 0.205	*l* = −32→32
28191 measured reflections	

### Refinement

**Table:**

Refinement on *F*^2^	Primary atom site location: structure-invariant direct methods
Least-squares matrix: full	Secondary atom site location: difference Fourier map
*R*[*F*^2^ > 2σ(*F*^2^)] = 0.033	Hydrogen site location: inferred from neighbouring sites
*wR*(*F*^2^) = 0.067	H-atom parameters constrained
*S* = 1.02	*w* = 1/[σ^2^(*F*_o_^2^) + (0.0236*P*)^2^ + 0.7178*P*] where *P* = (*F*_o_^2^ + 2*F*_c_^2^)/3
6825 reflections	(Δ/σ)_max_ = 0.002
353 parameters	Δρ_max_ = 0.64 e Å^−^^3^
4 restraints	Δρ_min_ = −0.63 e Å^−^^3^

### Special details

**Table:**

Geometry. Bond distances, angles *etc*. have been calculated using the rounded fractional coordinates. All su's are estimated from the variances of the (full) variance-covariance matrix. The cell e.s.d.'s are taken into account in the estimation of distances, angles and torsion angles
Refinement. Refinement of *F*^2^ against ALL reflections. The weighted *R*-factor *wR* and goodness of fit *S* are based on *F*^2^, conventional *R*-factors *R* are based on *F*, with *F* set to zero for negative *F*^2^. The threshold expression of *F*^2^ > σ(*F*^2^) is used only for calculating *R*-factors(gt) *etc*. and is not relevant to the choice of reflections for refinement. *R*-factors based on *F*^2^ are statistically about twice as large as those based on *F*, and *R*- factors based on ALL data will be even larger.

### Fractional atomic coordinates and isotropic or equivalent isotropic displacement parameters (Å^2^)

**Table:**

	*x*	*y*	*z*	*U*_iso_*/*U*_eq_	Occ. (<1)
Hg1	0.95842 (3)	0.35919 (1)	0.12435 (1)	0.0519 (1)	
Cl1	1.1843 (2)	0.24871 (10)	0.13636 (6)	0.0606 (6)	
Cl2	0.7153 (2)	0.26145 (9)	0.12825 (6)	0.0603 (6)	
S1	0.9833 (2)	0.43857 (9)	0.19776 (5)	0.0556 (6)	
S2	0.9520 (3)	0.39899 (10)	0.03940 (6)	0.0709 (7)	
N1	0.9163 (6)	0.3970 (3)	0.28644 (16)	0.0491 (19)	
N2	1.0045 (6)	0.2985 (3)	0.23990 (16)	0.0508 (19)	
N3	0.8804 (8)	0.3178 (3)	−0.04002 (18)	0.074 (3)	
N4	0.9391 (8)	0.2450 (3)	0.02721 (18)	0.075 (3)	
C1	0.9653 (7)	0.3727 (3)	0.24497 (19)	0.041 (2)	
C2	0.8766 (8)	0.4765 (3)	0.2998 (2)	0.060 (3)	
C3	1.0017 (10)	0.5085 (4)	0.3362 (3)	0.101 (4)	
C4	0.9988 (9)	0.2405 (3)	0.2782 (2)	0.058 (3)	
C5	1.0433 (10)	0.1618 (3)	0.2587 (3)	0.075 (3)	
C6	0.9220 (8)	0.3130 (4)	0.0071 (2)	0.053 (3)	
C7A	0.8161 (19)	0.3860 (9)	−0.0696 (5)	0.091 (4)	0.654 (17)
C8A	0.9587 (19)	0.4054 (8)	−0.0977 (6)	0.091 (4)	0.654 (17)
C9	0.9076 (10)	0.1702 (4)	0.0044 (2)	0.079 (3)	
C10	0.9186 (12)	0.1079 (5)	0.0400 (3)	0.117 (5)	
C8B	0.887 (4)	0.3928 (17)	−0.1187 (8)	0.091 (4)	0.346 (17)
C7B	0.898 (5)	0.3925 (13)	−0.0644 (8)	0.091 (4)	0.346 (17)
Hg2	0.45408 (4)	0.33101 (1)	0.37662 (1)	0.0591 (1)	
Cl3	0.7802 (2)	0.31660 (11)	0.38049 (6)	0.0708 (7)	
Cl4	0.3786 (3)	0.18733 (9)	0.37183 (6)	0.0722 (7)	
S3	0.3744 (3)	0.38683 (10)	0.45291 (6)	0.0766 (8)	
S4	0.3975 (2)	0.39347 (9)	0.29678 (5)	0.0522 (6)	
N5	0.4167 (8)	0.3390 (3)	0.54307 (18)	0.074 (2)	
N6	0.4005 (6)	0.2416 (3)	0.48716 (17)	0.0579 (19)	
N7	0.4886 (6)	0.2545 (3)	0.26517 (16)	0.0531 (19)	
N8	0.4977 (6)	0.3543 (2)	0.21149 (16)	0.0458 (19)	
C11	0.4011 (8)	0.3162 (3)	0.4975 (2)	0.051 (3)	
C12	0.4251 (11)	0.4203 (4)	0.5592 (3)	0.089 (4)	
C13	0.4601 (10)	0.4266 (4)	0.6119 (2)	0.096 (4)	
C14	0.4213 (9)	0.1796 (3)	0.5235 (2)	0.065 (3)	
C15	0.4173 (9)	0.1021 (4)	0.5003 (2)	0.076 (3)	
C16	0.4676 (7)	0.3284 (3)	0.25482 (19)	0.041 (2)	
C17	0.5442 (8)	0.1961 (3)	0.2317 (2)	0.056 (3)	
C18	0.5610 (10)	0.1179 (3)	0.2559 (2)	0.079 (3)	
C19	0.4814 (9)	0.4352 (3)	0.1962 (2)	0.059 (3)	
C20	0.4733 (9)	0.4443 (4)	0.1426 (2)	0.071 (3)	
H2	1.03531	0.28366	0.21217	0.0606*	
H1	0.90621	0.36223	0.30841	0.0589*	
H3	0.89178	0.27570	−0.05619	0.0882*	
H3A	1.10960	0.50984	0.32317	0.1513*	
H3B	1.00835	0.47640	0.36488	0.1513*	
H3C	0.96950	0.56010	0.34462	0.1513*	
H4	0.97321	0.24435	0.05771	0.0893*	
H2A	0.76690	0.47744	0.31258	0.0724*	
H2B	0.87090	0.50883	0.27084	0.0724*	
H5A	1.14476	0.16587	0.24245	0.1127*	
H5B	0.95328	0.14395	0.23609	0.1127*	
H5C	1.06001	0.12572	0.28526	0.1127*	
H7A	0.71729	0.37237	−0.09091	0.1097*	0.654 (17)
H7B	0.78855	0.42885	−0.04883	0.1097*	0.654 (17)
H8A	0.97447	0.36475	−0.12077	0.1372*	0.654 (17)
H8B	1.05873	0.41079	−0.07600	0.1372*	0.654 (17)
H8C	0.93610	0.45331	−0.11485	0.1372*	0.654 (17)
H9A	0.98916	0.16128	−0.01925	0.0947*	
H9B	0.79636	0.17016	−0.01287	0.0947*	
H10A	0.84951	0.12016	0.06588	0.1761*	
H10B	1.03338	0.10199	0.05310	0.1761*	
H10C	0.88015	0.06045	0.02457	0.1761*	
H4A	0.88679	0.23883	0.28949	0.0693*	
H4B	1.07778	0.25415	0.30563	0.0693*	
H7C	1.00573	0.41460	−0.05262	0.1097*	0.346 (17)
H7D	0.81128	0.42698	−0.05423	0.1097*	0.346 (17)
H8D	0.80269	0.35662	−0.13110	0.1372*	0.346 (17)
H8E	0.99427	0.37795	−0.12946	0.1372*	0.346 (17)
H8F	0.85848	0.44387	−0.13049	0.1372*	0.346 (17)
H5	0.42251	0.30364	0.56523	0.0885*	
H6	0.38699	0.22842	0.45693	0.0697*	
H7	0.46793	0.23940	0.29384	0.0635*	
H8	0.52933	0.32129	0.19069	0.0549*	
H12A	0.51277	0.44686	0.54330	0.1066*	
H12B	0.31894	0.44566	0.54947	0.1066*	
H13A	0.57405	0.41095	0.62097	0.1445*	
H13B	0.38440	0.39364	0.62778	0.1445*	
H13C	0.44482	0.47942	0.62178	0.1445*	
H14A	0.33199	0.18295	0.54522	0.0781*	
H14B	0.52781	0.18627	0.54281	0.0781*	
H15A	0.50405	0.09893	0.47822	0.1138*	
H15B	0.30953	0.09412	0.48262	0.1138*	
H15C	0.43555	0.06290	0.52497	0.1138*	
H17A	0.65194	0.21139	0.22075	0.0672*	
H17B	0.46363	0.19282	0.20327	0.0672*	
H18A	0.45433	0.10259	0.26656	0.1176*	
H18B	0.64300	0.12074	0.28348	0.1176*	
H18C	0.59652	0.08039	0.23307	0.1176*	
H19A	0.57666	0.46430	0.21090	0.0708*	
H19B	0.38008	0.45676	0.20803	0.0708*	
H20A	0.38707	0.41109	0.12766	0.1062*	
H20B	0.57989	0.43045	0.13128	0.1062*	
H20C	0.44772	0.49729	0.13419	0.1062*	

### Atomic displacement parameters (Å^2^)

**Table:**

	*U*^11^	*U*^22^	*U*^33^	*U*^12^	*U*^13^	*U*^23^
Hg1	0.0686 (2)	0.0563 (2)	0.0315 (1)	−0.0025 (1)	0.0075 (1)	−0.0046 (1)
Cl1	0.0633 (11)	0.0720 (12)	0.0466 (10)	0.0176 (8)	0.0054 (8)	−0.0071 (8)
Cl2	0.0650 (11)	0.0670 (11)	0.0510 (10)	−0.0129 (8)	0.0175 (9)	−0.0077 (8)
S1	0.0969 (13)	0.0385 (9)	0.0325 (9)	−0.0040 (9)	0.0125 (9)	−0.0008 (7)
S2	0.1290 (16)	0.0492 (11)	0.0345 (10)	−0.0123 (11)	0.0072 (10)	0.0021 (8)
N1	0.080 (4)	0.035 (3)	0.034 (3)	−0.001 (3)	0.015 (3)	0.000 (2)
N2	0.085 (4)	0.034 (3)	0.034 (3)	0.000 (3)	0.008 (3)	−0.003 (2)
N3	0.143 (6)	0.049 (4)	0.028 (3)	−0.002 (3)	−0.001 (3)	0.002 (3)
N4	0.146 (6)	0.048 (4)	0.028 (3)	0.000 (4)	−0.001 (3)	0.001 (3)
C1	0.061 (4)	0.034 (4)	0.029 (3)	0.000 (3)	0.004 (3)	−0.005 (3)
C2	0.101 (5)	0.037 (4)	0.045 (4)	0.005 (4)	0.020 (4)	−0.003 (3)
C3	0.145 (8)	0.057 (5)	0.095 (6)	−0.014 (5)	−0.022 (6)	−0.038 (5)
C4	0.093 (5)	0.032 (4)	0.049 (4)	0.001 (3)	0.009 (4)	0.005 (3)
C5	0.117 (6)	0.033 (4)	0.079 (5)	0.011 (4)	0.025 (5)	0.002 (4)
C6	0.070 (4)	0.059 (5)	0.032 (4)	−0.003 (3)	0.009 (3)	0.006 (3)
C7A	0.144 (11)	0.085 (5)	0.042 (5)	−0.015 (6)	−0.009 (6)	−0.008 (5)
C8A	0.144 (11)	0.085 (5)	0.042 (5)	−0.015 (6)	−0.009 (6)	−0.008 (5)
C9	0.137 (7)	0.053 (5)	0.046 (4)	0.007 (4)	−0.001 (4)	−0.006 (4)
C10	0.185 (10)	0.066 (6)	0.100 (7)	0.004 (6)	0.006 (7)	−0.003 (5)
C8B	0.144 (11)	0.085 (5)	0.042 (5)	−0.015 (6)	−0.009 (6)	−0.008 (5)
C7B	0.144 (11)	0.085 (5)	0.042 (5)	−0.015 (6)	−0.009 (6)	−0.008 (5)
Hg2	0.0898 (2)	0.0551 (2)	0.0336 (2)	−0.0059 (1)	0.0125 (1)	0.0014 (1)
Cl3	0.0791 (12)	0.0897 (14)	0.0445 (10)	0.0056 (10)	0.0111 (9)	0.0232 (9)
Cl4	0.1183 (15)	0.0552 (11)	0.0435 (10)	−0.0246 (10)	0.0089 (10)	0.0010 (8)
S3	0.1470 (18)	0.0465 (10)	0.0404 (10)	0.0170 (11)	0.0327 (11)	0.0069 (8)
S4	0.0836 (12)	0.0399 (9)	0.0336 (9)	0.0102 (8)	0.0088 (8)	−0.0016 (7)
N5	0.156 (6)	0.031 (3)	0.035 (3)	−0.010 (3)	0.011 (4)	0.000 (2)
N6	0.100 (4)	0.040 (3)	0.035 (3)	0.001 (3)	0.013 (3)	0.002 (3)
N7	0.096 (4)	0.035 (3)	0.029 (3)	0.009 (3)	0.010 (3)	0.005 (2)
N8	0.078 (4)	0.028 (3)	0.032 (3)	0.001 (2)	0.009 (3)	−0.001 (2)
C11	0.080 (5)	0.041 (4)	0.035 (4)	−0.006 (3)	0.017 (3)	0.001 (3)
C12	0.163 (8)	0.045 (5)	0.060 (5)	−0.018 (5)	0.017 (5)	−0.005 (4)
C13	0.172 (8)	0.060 (5)	0.059 (5)	−0.015 (5)	0.022 (5)	−0.019 (4)
C14	0.106 (6)	0.043 (4)	0.046 (4)	−0.003 (4)	0.009 (4)	0.003 (3)
C15	0.118 (6)	0.048 (5)	0.063 (5)	0.010 (4)	0.022 (4)	0.005 (4)
C16	0.054 (4)	0.038 (4)	0.029 (3)	0.002 (3)	−0.001 (3)	0.000 (3)
C17	0.083 (5)	0.039 (4)	0.047 (4)	0.010 (3)	0.010 (3)	−0.004 (3)
C18	0.129 (6)	0.039 (4)	0.072 (5)	0.011 (4)	0.030 (5)	0.003 (4)
C19	0.092 (5)	0.035 (4)	0.051 (4)	0.008 (3)	0.010 (4)	0.006 (3)
C20	0.101 (5)	0.058 (5)	0.055 (5)	0.015 (4)	0.014 (4)	0.025 (4)

### Geometric parameters (Å, °)

**Table:**

Hg1—Cl1	2.6220 (17)	C3—H3C	0.9600
Hg1—Cl2	2.5767 (16)	C4—H4B	0.9700
Hg1—S1	2.4335 (15)	C4—H4A	0.9700
Hg1—S2	2.4323 (17)	C5—H5C	0.9600
Hg2—Cl4	2.5487 (16)	C5—H5B	0.9600
Hg2—S3	2.4415 (18)	C5—H5A	0.9600
Hg2—S4	2.4534 (14)	C7A—H7A	0.9700
Hg2—Cl3	2.6046 (16)	C7A—H7B	0.9700
S1—C1	1.740 (5)	C7B—H7D	0.9700
S2—C6	1.734 (7)	C7B—H7C	0.9700
S3—C11	1.728 (6)	C8A—H8B	0.9600
S4—C16	1.736 (5)	C8A—H8C	0.9600
N1—C2	1.460 (7)	C8A—H8A	0.9600
N1—C1	1.306 (7)	C8B—H8E	0.9600
N2—C1	1.326 (7)	C8B—H8F	0.9600
N2—C4	1.456 (7)	C8B—H8D	0.9600
N3—C6	1.313 (7)	C9—H9B	0.9700
N3—C7A	1.495 (16)	C9—H9A	0.9700
N3—C7B	1.46 (2)	C10—H10B	0.9600
N4—C6	1.298 (8)	C10—H10C	0.9600
N4—C9	1.446 (8)	C10—H10A	0.9600
N1—H1	0.8600	C12—C13	1.457 (10)
N2—H2	0.8600	C14—C15	1.479 (8)
N3—H3	0.8600	C17—C18	1.504 (7)
N4—H4	0.8600	C19—C20	1.479 (8)
N5—C12	1.469 (9)	C12—H12A	0.9700
N5—C11	1.310 (7)	C12—H12B	0.9700
N6—C14	1.463 (7)	C13—H13A	0.9600
N6—C11	1.317 (7)	C13—H13B	0.9600
N7—C16	1.313 (7)	C13—H13C	0.9600
N7—C17	1.458 (7)	C14—H14A	0.9700
N8—C19	1.459 (6)	C14—H14B	0.9700
N8—C16	1.314 (7)	C15—H15A	0.9600
N5—H5	0.8600	C15—H15B	0.9600
N6—H6	0.8600	C15—H15C	0.9600
N7—H7	0.8600	C17—H17A	0.9700
N8—H8	0.8600	C17—H17B	0.9700
C2—C3	1.460 (10)	C18—H18A	0.9600
C4—C5	1.512 (8)	C18—H18B	0.9600
C7A—C8A	1.47 (2)	C18—H18C	0.9600
C7B—C8B	1.49 (3)	C19—H19A	0.9700
C9—C10	1.451 (11)	C19—H19B	0.9700
C2—H2A	0.9700	C20—H20A	0.9600
C2—H2B	0.9700	C20—H20B	0.9600
C3—H3B	0.9600	C20—H20C	0.9600
C3—H3A	0.9600		
			
Hg1···H2	2.7700	H4···H10A	2.3700
Hg1···H20B	3.2800	H4···H10B	2.5000
Hg1···H4	2.7100	H4···Cl2	2.9600
Hg1···H8C^i^	3.3500	H4···Cl1	2.6300
Hg1···H8F^i^	3.6900	H4A···H17A	2.5900
Hg1···H20A^ii^	3.5300	H4A···Cl3	3.0200
Hg2···H6	2.9100	H4A···N1	2.7400
Hg2···H7	2.7800	H4A···H1	2.1900
Hg2···H4B^iii^	3.6800	H4B···Cl4^ii^	3.1100
Hg2···H13C^iv^	3.3600	H4B···N1	2.8100
Cl1···N4	3.442 (6)	H4B···Hg2^ii^	3.6800
Cl1···N2	3.406 (5)	H4B···H1	2.3200
Cl2···C17	3.448 (6)	H5···C14	2.4300
Cl2···N4	3.440 (6)	H5···H13B	2.3600
Cl2···N8	3.390 (5)	H5···Cl2^v^	3.0000
Cl3···C9^v^	3.481 (6)	H5···H14B	2.2900
Cl3···N1	3.205 (5)	H5···H14A	2.2500
Cl3···N3^v^	3.239 (5)	H5···Cl1^xii^	2.9800
Cl4···N7	3.340 (5)	H5A···H2	2.3300
Cl4···N6	3.299 (5)	H5B···H2	2.5900
Cl1···H13B^vi^	2.9500	H5C···C8B^v^	3.1000
Cl1···H2	2.5600	H5C···H8E^v^	2.4500
Cl1···H4	2.6300	H6···Hg2	2.9100
Cl1···H17B^ii^	2.9300	H6···Cl4	2.4400
Cl1···H5^vi^	2.9800	H6···H15A	2.4700
Cl1···H14A^vi^	3.0900	H6···H15B	2.5100
Cl2···H20B	3.1100	H7···Cl4	2.4900
Cl2···H17A	2.7700	H7···H18A	2.4700
Cl2···H4	2.9600	H7···H18B	2.5100
Cl2···H14B^vii^	2.8200	H7···Hg2	2.7800
Cl2···H8	2.5800	H7A···Cl4^vii^	2.9900
Cl2···H5^vii^	3.0000	H7B···H15A^vii^	2.4900
Cl3···H9B^v^	2.9300	H7B···S2	2.7100
Cl3···H8D^v^	3.0100	H7C···H15B^vi^	2.5400
Cl3···H1	2.4300	H7C···S2	2.6200
Cl3···H4A	3.0200	H7D···S2	2.7600
Cl3···H9A^v^	3.1200	H8···H17B	2.3100
Cl3···H3^v^	2.4700	H8···H17A	2.2600
Cl4···H7	2.4900	H8···H20B	2.5500
Cl4···H7A^v^	2.9900	H8···C17	2.4300
Cl4···H4B^iii^	3.1100	H8···Cl2	2.5800
Cl4···H6	2.4400	H8···H20A	2.5200
S4···C1^iii^	3.633 (6)	H8A···H3	2.4800
S1···H2B	2.5700	H8B···H15B^vi^	2.4600
S1···H8C^i^	3.0600	H8C···S1^i^	3.0600
S1···H19B^ii^	3.1700	H8C···Hg1^i^	3.3500
S1···H8F^i^	3.0800	H8D···Cl3^vii^	3.0100
S2···H7B	2.7100	H8D···H3	2.5400
S2···H7C	2.6200	H8E···H5C^vii^	2.4500
S2···H7D	2.7600	H8F···S1^i^	3.0800
S3···H12A	2.8300	H8F···Hg1^i^	3.6900
S3···H12B	2.9100	H9A···N3	2.8800
S3···H12A^iv^	3.0000	H9A···H3	2.3200
S4···H19B	2.6700	H9A···Cl3^vii^	3.1200
S4···H3A^iii^	3.1800	H9B···Cl3^vii^	2.9300
S4···H19A	3.1100	H9B···N3	2.7500
N1···Cl3	3.205 (5)	H9B···H3	2.3400
N2···Cl1	3.406 (5)	H10A···H4	2.3700
N3···Cl3^vii^	3.239 (5)	H10B···H4	2.5000
N4···Cl1	3.442 (6)	H10B···H12B^vi^	2.4300
N4···Cl2	3.440 (6)	H12A···S3	2.8300
N6···Cl4	3.299 (5)	H12A···S3^iv^	3.0000
N7···Cl4	3.340 (5)	H12B···S3	2.9100
N8···Cl2	3.390 (5)	H12B···H10B^xii^	2.4300
N1···H4B	2.8100	H13B···H5	2.3600
N1···H4A	2.7400	H13B···Cl1^xii^	2.9500
N3···H9B	2.7500	H13B···H17B^v^	2.5900
N3···H9A	2.8800	H13C···Hg2^iv^	3.3600
N5···H14B	2.7800	H14A···N5	2.7700
N5···H14A	2.7700	H14A···H5	2.2500
N8···H17B	2.8000	H14A···Cl1^xii^	3.0900
N8···H17A	2.7500	H14B···N5	2.7800
C1···S4^ii^	3.633 (6)	H14B···H5	2.2900
C9···Cl3^vii^	3.481 (6)	H14B···Cl2^v^	2.8200
C17···Cl2	3.448 (6)	H15A···H6	2.4700
C5···H2B^viii^	2.8600	H15A···C7A^v^	2.9200
C7A···H15A^vii^	2.9200	H15A···H7B^v^	2.4900
C8B···H5C^vii^	3.1000	H15B···H6	2.5100
C9···H3	2.4600	H15B···H8B^xii^	2.4600
C14···H5	2.4300	H15B···H7C^xii^	2.5400
C17···H8	2.4300	H17A···Cl2	2.7700
C18···H19B^ix^	2.9700	H17A···N8	2.7500
C18···H19A^ix^	3.0400	H17A···H4A	2.5900
C19···H18A^x^	3.0900	H17A···H8	2.2600
H1···H3B	2.5900	H17B···Cl1^iii^	2.9300
H1···H4A	2.1900	H17B···N8	2.8000
H1···Cl3	2.4300	H17B···H8	2.3100
H1···C4	2.4000	H17B···H13B^vii^	2.5900
H1···H4B	2.3200	H18A···H7	2.4700
H2···Cl1	2.5600	H18A···C19^ix^	3.0900
H2···H5A	2.3300	H18A···H19A^ix^	2.4800
H2···H5B	2.5900	H18B···H7	2.5100
H2···Hg1	2.7700	H19A···S4	3.1100
H2B···C5^xi^	2.8600	H19A···C18^x^	3.0400
H2B···S1	2.5700	H19A···H18A^x^	2.4800
H3···H9A	2.3200	H19B···S1^iii^	3.1700
H3···C9	2.4600	H19B···S4	2.6700
H3···H8D	2.5400	H19B···C18^x^	2.9700
H3···H8A	2.4800	H20A···Hg1^iii^	3.5300
H3···H9B	2.3400	H20A···H8	2.5200
H3···Cl3^vii^	2.4700	H20B···Hg1	3.2800
H3A···S4^ii^	3.1800	H20B···Cl2	3.1100
H3B···H1	2.5900	H20B···H8	2.5500
H4···Hg1	2.7100		
			
Cl1—Hg1—Cl2	91.78 (5)	N3—C7B—H7D	108.00
Cl1—Hg1—S1	107.27 (5)	C8B—C7B—H7C	108.00
Cl1—Hg1—S2	106.50 (6)	H7C—C7B—H7D	107.00
Cl2—Hg1—S1	109.97 (5)	C8B—C7B—H7D	108.00
Cl2—Hg1—S2	105.65 (6)	H8A—C8A—H8B	109.00
S1—Hg1—S2	129.25 (5)	C7A—C8A—H8C	109.00
Cl4—Hg2—S3	110.47 (6)	C7A—C8A—H8A	109.00
Cl4—Hg2—S4	110.81 (5)	C7A—C8A—H8B	110.00
S3—Hg2—S4	123.74 (6)	H8A—C8A—H8C	109.00
Cl3—Hg2—S4	100.69 (5)	H8B—C8A—H8C	110.00
Cl3—Hg2—Cl4	98.04 (7)	H8E—C8B—H8F	109.00
Cl3—Hg2—S3	109.51 (7)	C7B—C8B—H8F	110.00
Hg1—S1—C1	104.29 (18)	C7B—C8B—H8E	109.00
Hg1—S2—C6	104.0 (2)	C7B—C8B—H8D	110.00
Hg2—S3—C11	107.9 (2)	H8D—C8B—H8E	109.00
Hg2—S4—C16	105.49 (19)	H8D—C8B—H8F	110.00
C1—N1—C2	127.5 (5)	N4—C9—H9A	109.00
C1—N2—C4	124.3 (5)	C10—C9—H9A	109.00
C6—N3—C7A	129.3 (7)	C10—C9—H9B	109.00
C6—N3—C7B	118.8 (11)	H9A—C9—H9B	108.00
C6—N4—C9	127.7 (5)	N4—C9—H9B	109.00
C2—N1—H1	116.00	C9—C10—H10C	109.00
C1—N1—H1	116.00	C9—C10—H10B	109.00
C4—N2—H2	118.00	C9—C10—H10A	109.00
C1—N2—H2	118.00	H10B—C10—H10C	110.00
C6—N3—H3	115.00	H10A—C10—H10B	109.00
C7B—N3—H3	119.00	H10A—C10—H10C	110.00
C7A—N3—H3	115.00	S3—C11—N5	117.7 (4)
C6—N4—H4	116.00	S3—C11—N6	122.4 (4)
C9—N4—H4	116.00	N5—C11—N6	119.9 (5)
C11—N5—C12	125.0 (5)	N5—C12—C13	111.8 (6)
C11—N6—C14	124.6 (5)	N6—C14—C15	111.6 (5)
C16—N7—C17	124.9 (5)	S4—C16—N7	121.7 (4)
C16—N8—C19	124.6 (4)	S4—C16—N8	118.6 (4)
C11—N5—H5	117.00	N7—C16—N8	119.8 (5)
C12—N5—H5	118.00	N7—C17—C18	111.1 (5)
C11—N6—H6	118.00	N8—C19—C20	112.6 (5)
C14—N6—H6	118.00	N5—C12—H12A	109.00
C17—N7—H7	118.00	N5—C12—H12B	109.00
C16—N7—H7	118.00	C13—C12—H12A	109.00
C19—N8—H8	118.00	C13—C12—H12B	109.00
C16—N8—H8	118.00	H12A—C12—H12B	108.00
N1—C1—N2	119.4 (5)	C12—C13—H13A	110.00
S1—C1—N2	121.0 (4)	C12—C13—H13B	110.00
S1—C1—N1	119.5 (4)	C12—C13—H13C	110.00
N1—C2—C3	112.2 (5)	H13A—C13—H13B	109.00
N2—C4—C5	109.7 (5)	H13A—C13—H13C	109.00
N3—C6—N4	119.1 (6)	H13B—C13—H13C	109.00
S2—C6—N3	117.7 (5)	N6—C14—H14A	109.00
S2—C6—N4	123.2 (4)	N6—C14—H14B	109.00
N3—C7A—C8A	102.8 (11)	C15—C14—H14A	109.00
N3—C7B—C8B	117.6 (19)	C15—C14—H14B	109.00
N4—C9—C10	111.6 (5)	H14A—C14—H14B	108.00
H2A—C2—H2B	108.00	C14—C15—H15A	110.00
C3—C2—H2B	109.00	C14—C15—H15B	109.00
N1—C2—H2A	109.00	C14—C15—H15C	109.00
N1—C2—H2B	109.00	H15A—C15—H15B	109.00
C3—C2—H2A	109.00	H15A—C15—H15C	109.00
H3A—C3—H3B	109.00	H15B—C15—H15C	109.00
C2—C3—H3C	110.00	N7—C17—H17A	109.00
C2—C3—H3B	109.00	N7—C17—H17B	109.00
C2—C3—H3A	110.00	C18—C17—H17A	109.00
H3B—C3—H3C	109.00	C18—C17—H17B	109.00
H3A—C3—H3C	110.00	H17A—C17—H17B	108.00
N2—C4—H4A	110.00	C17—C18—H18A	109.00
C5—C4—H4A	110.00	C17—C18—H18B	109.00
C5—C4—H4B	110.00	C17—C18—H18C	109.00
N2—C4—H4B	110.00	H18A—C18—H18B	110.00
H4A—C4—H4B	108.00	H18A—C18—H18C	109.00
H5B—C5—H5C	109.00	H18B—C18—H18C	109.00
H5A—C5—H5C	110.00	N8—C19—H19A	109.00
C4—C5—H5A	109.00	N8—C19—H19B	109.00
H5A—C5—H5B	110.00	C20—C19—H19A	109.00
C4—C5—H5C	109.00	C20—C19—H19B	109.00
C4—C5—H5B	109.00	H19A—C19—H19B	108.00
N3—C7A—H7A	111.00	C19—C20—H20A	109.00
N3—C7A—H7B	111.00	C19—C20—H20B	109.00
C8A—C7A—H7A	111.00	C19—C20—H20C	109.00
C8A—C7A—H7B	111.00	H20A—C20—H20B	110.00
H7A—C7A—H7B	109.00	H20A—C20—H20C	110.00
N3—C7B—H7C	108.00	H20B—C20—H20C	110.00
			
Cl1—Hg1—S1—C1	−54.2 (2)	C1—N1—C2—C3	−110.2 (7)
Cl2—Hg1—S1—C1	44.3 (2)	C4—N2—C1—S1	177.6 (5)
S2—Hg1—S1—C1	176.2 (2)	C1—N2—C4—C5	177.3 (6)
Cl1—Hg1—S2—C6	52.9 (2)	C4—N2—C1—N1	−0.7 (9)
Cl2—Hg1—S2—C6	−43.8 (2)	C6—N3—C7A—C8A	109.6 (11)
S1—Hg1—S2—C6	−177.2 (2)	C7A—N3—C6—N4	164.7 (9)
S4—Hg2—S3—C11	168.7 (2)	C7A—N3—C6—S2	−15.5 (12)
Cl3—Hg2—S3—C11	−73.1 (2)	C9—N4—C6—S2	176.0 (6)
Cl4—Hg2—S3—C11	33.8 (3)	C9—N4—C6—N3	−4.2 (11)
S3—Hg2—S4—C16	−176.8 (2)	C6—N4—C9—C10	−172.1 (7)
Cl3—Hg2—S4—C16	60.9 (2)	C12—N5—C11—S3	−4.5 (10)
Cl4—Hg2—S4—C16	−42.0 (2)	C12—N5—C11—N6	177.7 (7)
Hg1—S1—C1—N1	−156.7 (4)	C11—N5—C12—C13	−174.0 (7)
Hg1—S1—C1—N2	25.0 (5)	C14—N6—C11—S3	−179.5 (5)
Hg1—S2—C6—N3	166.9 (5)	C14—N6—C11—N5	−1.9 (10)
Hg1—S2—C6—N4	−13.3 (6)	C11—N6—C14—C15	−179.2 (6)
Hg2—S3—C11—N5	159.2 (5)	C17—N7—C16—S4	178.9 (4)
Hg2—S3—C11—N6	−23.1 (6)	C17—N7—C16—N8	−0.1 (9)
Hg2—S4—C16—N7	20.1 (5)	C16—N7—C17—C18	177.9 (6)
Hg2—S4—C16—N8	−160.9 (4)	C19—N8—C16—S4	2.0 (8)
C2—N1—C1—S1	−1.8 (8)	C19—N8—C16—N7	−179.0 (5)
C2—N1—C1—N2	176.5 (5)	C16—N8—C19—C20	−163.0 (6)

Symmetry codes: (i) −*x*+2, −*y*+1, −*z*; (ii) *x*+1, *y*, *z*; (iii) *x*−1, *y*, *z*; (iv) −*x*+1, −*y*+1, −*z*+1; (v) *x*, −*y*+1/2, *z*+1/2; (vi) *x*+1, −*y*+1/2, *z*−1/2; (vii) *x*, −*y*+1/2, *z*−1/2; (viii) −*x*+2, *y*−1/2, −*z*+1/2; (ix) −*x*+1, *y*−1/2, −*z*+1/2; (x) −*x*+1, *y*+1/2, −*z*+1/2; (xi) −*x*+2, *y*+1/2, −*z*+1/2; (xii) *x*−1, −*y*+1/2, *z*+1/2.

### Hydrogen-bond geometry (Å, °)

**Table:**

*D*—H···*A*	*D*—H	H···*A*	*D*···*A*	*D*—H···*A*
N1—H1···Cl3	0.86	2.43	3.205 (5)	150
N2—H2···Cl1	0.86	2.56	3.406 (5)	169
N3—H3···Cl3^vii^	0.86	2.47	3.239 (5)	149
N4—H4···Cl1	0.86	2.63	3.442 (6)	159
N4—H4···Cl2	0.86	2.96	3.440 (6)	117
N6—H6···Cl4	0.86	2.44	3.299 (5)	174
N7—H7···Cl4	0.86	2.49	3.340 (5)	173
N8—H8···Cl2	0.86	2.58	3.390 (5)	158
C14—H14B···Cl2^v^	0.97	2.82	3.699 (6)	151
C17—H17A···Cl2	0.97	2.77	3.448 (6)	127

Symmetry codes: (vii) *x*, −*y*+1/2, *z*−1/2; (v) *x*, −*y*+1/2, *z*+1/2.
